# What are the financial barriers to medical care among the poor, the sick and the disabled in the Special Administrative Region of China?

**DOI:** 10.1371/journal.pone.0205794

**Published:** 2018-11-14

**Authors:** Samuel Yeung-shan Wong, Roger Yat-nork Chung, Dicken Chan, Gary Ka-ki Chung, Jerry Li, Dominic Mak, Maggie Lau, Vera Tang, David Gordon, Hung Wong

**Affiliations:** 1 The Jockey Club School of Public Health and Primary Care, The Chinese University of Hong Kong, Hong Kong SAR, People’s Republic of China; 2 Faculty of Medicine, The Chinese University of Hong Kong, Hong Kong SAR, People’s Republic of China; 3 Asia-Pacific Institute of Ageing Studies, Lingnan University, Hong Kong SAR, People’s Republic of China; 4 School for Policy Studies, University of Bristol, Bristol, United Kingdom; 5 Department of Social Work, The Chinese University of Hong Kong, Hong Kong SAR, People’s Republic of China; Indiana University Purdue University at Indianapolis, UNITED STATES

## Abstract

Although Hong Kong is one of the richest cities in the world and has some of the best health outcomes such as long life expectancy, little is known about the people who are unable to access healthcare due to lack of financial means. Cross-sectional data from a sample of 2,233 participants aged 18 or above was collected from the first wave of the “Trends and Implications of Poverty and Social Disadvantages in Hong Kong” survey. Socio-demographic factors, lifestyle factors, and physical and mental health conditions associated with people who were unable to seek medical services due to lack of financial means in the past year were examined using forward stepwise logistic regression analyses. Of the 2,233 participants surveyed, 8.4% did not seek medical care due to lack of financial means during the past year. They were more likely to be income-poor. With respect to physical and mental health, despite having less likelihood to have multimorbidity, they tended to have higher levels of both anxiety and stress, poorer physical and mental health-related quality of life, and suffer from more severe disability and pain symptoms affecting their daily activities, when compared to the rest of the Hong Kong population. People who were denied of medical care due to financial barriers are generally sicker than people in the general Hong Kong population, implying that those with greater healthcare needs may have financial difficulties in receiving timely and appropriate medical care. Our findings suggest that inequity in healthcare utilization remains a critical issue in Hong Kong.

## Introduction

Universal health coverage (UHC) is defined by the World Health Organization (WHO) as “universal access to needed health services without financial hardship in paying for them”[1]. Since the passage of the United Nations General Assembly resolution on UHC in 2012, the development of health systems to provide UHC has become a central health goal for many countries, both as part of the Millennium Development Goal framework and the more recent Sustainable Development Goal 3 to “achieve universal health coverage, including financial risk protection”[2]. To measure progress towards UHC, timely assessment and monitoring of population access and coverage for essential health services are needed[3]. Hong Kong, a prior British colony and now a Special Administrative Region of China, is an example of a mixed public and private healthcare system where the bulk of outpatient services (70%) are delivered by the private sector (usually with patients paying out-of-pocket) while more than 90% of inpatient services are provided by the public sector[4]. Some low-income people, who meet the eligibility requirements, can receive financial assistance from the Comprehensive Social Security Assistance (CSSA) Scheme with free medical treatment at public hospitals or clinics in Hong Kong. The rest of the population can utilize public medical services paying a nominal fee of approximately US $6 for primary outpatient care services, US $8 for specialist outpatient services, and US $12 for the daily rate of hospitalization[5]. One of the well-recognized features of the Hong Kong healthcare system is that it is one of the most efficient systems in the world although this likely refers only to the public sector[6]. The Hong Kong population has one of the longest life expectancies[7] and one of the lowest neonatal mortality rates in the world, despite relatively low government expenditure on healthcare (about 5·4% of GDP)[8].

Although the Hong Kong population has achieved excellent health outcomes, little is known about people who are unable to access healthcare services due to lack of financial means, especially given a widening income gap between the rich and the poor over recent decades in Hong Kong[9]. Despite the presence of a tax-funded public healthcare sector with the policy goal to ensure that no one is denied adequate medical treatment due to financial reasons[10], a previous local study suggested a mismatch between healthcare needs and service utilization among older adults in Hong Kong[11]. In light of this, we aim to assess the inequalities in healthcare utilization among community-dwelling adults in Hong Kong. Specifically, the objectives of this research are 1) to describe the proportion of people in Hong Kong who were unable to access healthcare due to lack of financial means; and 2) to examine the medical and lifestyle characteristics of this population and assess independent demographic and health related factors for this group.

## Methods

### Subjects and methods

The data used in this study came from the first-wave survey of the “Trends and Implications of Poverty and Social Disadvantages in Hong Kong: A Multi-disciplinary and Longitudinal Study” (SPPR Research) that was conducted by the Social Disadvantages, Well-being and Health in Hong Kong (SDWH-HK) research team between June 2014 and August 2015. The data were collected from a random sample of all households in Hong Kong through face-to-face interview. The sample was drawn from 25,000 addresses and 200 segments by the Census and Statistics Department based on the frame of quarters. A two-stage stratified sample design was adopted, with the records in the frame of quarters first stratified by geographical area (i.e., the respondents' living areas by District Council Districts) and then by type of living quarters (i.e., public and private housing). For the quarters selected, all households residing in them were selected for inclusion in the survey. In the second stage, a respondent aged 18 years or above within each household was recruited to answer the adult questionnaire, and if the household had more than one adult, the one whose birthday was coming up next would be selected. A household was defined as people living alone or living together in the same quarter, but not necessarily related, who make common provision for essentials for living. In this study, 4,947 addresses were sampled of which 3,791 were valid. The final sample consisted of 2,282 household respondents aged 18 years or above; i.e., the response rate was 60·2%. A total of 2,233 questionnaires with valid data were included in this analysis. The interview was based on a structured questionnaire, and information on socio-demographic factors, lifestyle factors, and physical and mental health conditions were collected by trained professional interviewers.

#### Socio-demographic factors

Marital status was recorded as either married (including cohabitation) or unmarried (including never married, divorced, separated, or widowed). Education level was categorized as primary or below, secondary, and tertiary or above. The classification of occupation of respondent’s current or last jobs was based on the four International Standard Classification of Occupation 2008 (ISCO-08) skill levels of the International Labour Organization[12] (Skill levels 3 or 4: Managers and administrators / Professionals / Associate professionals, Skill level 2: Clerical support workers / Service and sales workers / Craft and related workers / Plant and machine operators and assemblers, Skill level 1: Elementary occupations / Others). Respondents who were students and persons looking after family / home were also included in the analysis. The official poverty line of Hong Kong was defined as half of the median monthly household income of all domestic households prior to government intervention. This income-based approach adopted the concept of relative poverty which would move with the times and change with general living standards. The 50% benchmark has been widely quoted and well recognised where Organization for Economic Cooperation and Development (OECD) adopted it as their headline poverty threshold. In our study, participants were asked to estimate their total pre-tax monthly household income including social security benefits from pre-defined income bands. The midpoint of each income band was assigned to represent continuous income in that category. Equivalised household income was derived by dividing household income by the square root of household size to allow for economies of scale when comparing different sized households[13]. People whose equivalised monthly household incomes fell below half of the median equivalised household monthly income in our samples were classified as “Poor.”

#### Lifestyle factors

Smoking status was defined as non-smoker and past or current smoker. Alcohol drinking was classified in terms of non-risky drinker and risky drinker, using the Alcohol Use Disorders Identification Test–Consumption (AUDIT-C)[14]. AUDIT-C is an abbreviated version of the full AUDIT questionnaire, which is derived from the first three questions of the 10-question AUDIT instrument. Each AUDIT-C question was scored from 0 to 4 points and questions were summed to a total AUDIT-C score of 0 to 12 points. For populations with a low prevalence of alcohol abuse and dependence such as Hong Kong, a respondent with a score of five points or more was identified as a risky drinker who was potentially drinking at increasing risk or higher risk levels[15]. Level of physical activity was assessed by the short form of the International Physical Activity Questionnaire (IPAQ) designed for population surveillance of physical activity among adults[16]. The short form recorded time spent per week in vigorous-intensity activity, moderate-intensity activity, and walking. Total weekly physical activity was estimated by multiplying reported minutes per week within each activity category by the Metabolic Equivalent of Task (MET) level assigned to each category of activity. Three levels of physical activity (active, minimally active, and inactive) were used for classification.

#### Physical and mental health conditions

Medical history of chronic diseases was collected based on respondents’ reports of conditions diagnosed by a doctor. Self-reporting of chronic diseases has been considered as a valid and reliable method for collecting medical diagnosis in large-scale study [17]. Self-perception of weight is often independent of objective body size and differed from public health standard. Participants were asked to describe their body image using five response options, “very thin,” “a little bit thin,” “about right,” “a little bit fat” and “very fat” [18]. Respondents were considered as having negative body image if they described themselves as not being “about right.” We used one question from the Pittsburgh Sleep Quality Index (PSQI)[19] to rate participants’ overall sleep quality. Four-response categories of perceived sleep quality were collapsed into two groups (very good/fairly good and very bad/fairly bad) to discriminate the “good” and the “poor” sleepers. In accordance with the chronic pain grade (CPG) questionnaire [20], the participants were asked the extent to which daily activities were affected by pain, which was captured in three categories: no pain at all, not affected/a little bit affected, and quite affected/extremely affected.

Regarding the anthropometric indicators, resting blood pressure was measured twice with participants being in a sitting position using automatic blood pressure monitors (Medel Elite, model number: COD. 92125), the first time at 15 minutes after starting the interview and the second time before the end of the interview. The average reading of blood pressure was used for the analysis. High blood pressure was defined as having systolic blood pressure ≥ 140mmHg and/or diastolic blood pressure ≥ 90 mmHg[21]. For height measurement, participants were asked to stand upright against a wall without shoes and look straight ahead, and their standing heights were measured to the nearest 0·1 cm using a standard tape measure. Body weight was measured to the nearest 0·1 kg with the participants wearing light indoor clothing without shoes using portable digital weight scales. Body mass index (BMI) was calculated as weight in kilograms divided by height in meters squared (kg/m^2^). Participants were classified as normal-weight (BMI of 18·5–22·9), overweight (BMI of 23·0–24·9), obese (BMI ≥ 25), or underweight (BMI < 18·5). The waist circumference was measured to the nearest 0·1 cm by midway between the lower borders of rib cage and the top of the iliac crest. Participants with waist circumference larger than the cut-off values for South Asians (≥ 90 cm for men, ≥ 80 cm for women) would be regarded to have “central obesity”[22].

To assess the extent of disability, a set of six core screening questions, proposed by the United Nations Washington Group on Disability Statistics[23], was adopted. Six questions from the short-set questionnaire were designed to identify respondents who had functional limitation in a core set of basic activities including vision, hearing, mobility, remembering, self-care, and communicating. Two prevalence rates were calculated to indicate the multidimensionality of disability, the presence of at least some difficulty in any of the six domains (P1) and at least some difficulties on at least two of the six domains (PM, where M stands for multiple domains).

Health-related quality of life was assessed using the Medical Outcomes Study 12-item Short-Form Survey version 2 (SF-12 v2), which has been validated for the Hong Kong Chinese population[24]. The instrument covers eight domains including physical functioning, role physical, bodily pain, general health, vitality, social functioning, role emotional, and mental health. Although the SF-12 v2 provided estimates of all eight domains, we focused on the two summary scores, Physical Component Summary (PCS) and Mental Component Summary (MCS), which are derived from all domains. We applied a norm-based scoring algorithm with reference to the data from a Hong Kong general population survey[25]. The first question of the SF-12 v2 was selected to evaluate self-rated health according to respondent’s own definition of health. Participants were assumed as healthy and did not need to seek medical care in the past year if they reported themselves as excellent or very good in self-rated health.

Anxiety and stress levels were measured using the subscales of the Depression Anxiety Stress Scales (DASS-21) which is a validated 21-item self-reporting questionnaire[26]. Participants were asked to rate how much the statement applied to them over the past week based on a four-point rating scale, ranging from 0 (did not apply to the respondent at all) to 3 (applied to the respondent very much, or most of the time). To calculate comparable scores with full DASS-42, subscales of anxiety and stress were derived by summing the scores for each subscale and multiplying by two. Each subscale has standardized clinical cutoffs (anxiety: normal (0–7), mild (8–9), moderate (10–14), severe (15–19), extremely severe (≥20); stress: normal (0–14), mild (15–18), moderate (19–25), severe (26–33), extremely severe (≥34)). Subscales of DASS have been found to be valid and reliable amongst Asian populations[27]. Respondents who obtained a score of mild to extremely severe levels were regarded as anxious or stressed.

#### Primary outcome–medical access

All participants were asked “In the past year, has a lack of money prevented you from seeing a doctor?” with a “Yes/No” dichotomous response. This was the primary outcome of the study.

### Statistical analysis

Continuous variables are presented as mean ± standard deviation (SD) and categorical variables as count and percentage. Compared with demographics of the general population of Hong Kong, participants in this study were older and had lower educational levels. To ensure the representativeness of the results, the raw data were weighted by Hong Kong resident population by age and sex at mid-2014. All figures presented were based on the weighted sample. Physical and mental component summary scores derived from SF-12 v2 were stratified into quartiles for our analyses. The association between medical services access and their correlates were analysed using univariate and multivariable analyses. For each independent variable, odds ratios (ORs) and 95% confidence intervals (CIs) were obtained. All significant variables in the primary univariate analyses were further examined using forward stepwise logistic regression. The statistical package SPSS version 21 (SPSS Inc., Illinois, US) was employed for all statistical analyses. All statistical tests were two-tailed with a significant level of 0·05. Confidence intervals are provided wherever appropriate.

## Results

The original and weighted basic characteristics of the respondents are presented in Table 1.

**Table 1:** Basic characteristics of the respondents (population weighted by age and sex).

	Original %	Weighted % ^a^
Age (year)		
18 ~	12.0	18.3
30 ~	12.0	18.3
40 ~	20.6	18.8
50 ~	18.9	20.1
60 ~	17.9	12.6
70 ~	11.4	6.8
80 ~	7.2	5.1
Gender		
Male	40.7	45.3
Female	59.3	54.7
Marital status		
Married/ Cohabit	62.8	61.7
Single / Divorced / Separated / Widowed	37.2	38.3
Education level		
Primary or below	33.3	25.4
Secondary	52.2	55.1
Tertiary or above	14.4	19.4
Occupation		
Skill levels 3 or 4	11.8	14.4
Skill level 2	37.2	39.5
Skill level 1	22.5	19.4
Student	5.1	5.4
Looking after family / home	23.4	21.3

^a^ Weighted according to the census data in terms of age and gender.

The characteristics of participants who were unable to access healthcare due to lack of financial means are shown in Table 2. Of the 2,233 respondents who answered the question about medical service access, 8·4% stated that they were prevented from seeking medical care due to lack of financial means in the past year. This population was also more likely to be female, minimally educated, less skilled or non-employed, income-poor. Despite being minimally active was significantly less likely to be associated with having financial barriers to medical care, there was no clear trend across the physical activity levels. With respect to physical and mental health, they tended to have central obesity and a negative body image, suffer from pain symptoms that affected their daily activities, disability, and multi-morbidity, and reported poorer sleep quality, higher levels of both anxiety and stress, and worse physical and mental health-related quality of life, when compared with the rest of the Hong Kong population (i.e., those who were not prevented from seeking medical care due to lack of financial means).

**Table 2:** Crude odds ratio for having financial barriers to medical care in relation to socio-demographic factors, lifestyle factors, and physical and mental health conditions.

	Row %	Crude OR	95% CI
**Socio-demographic factors**			
Age (years)		1.00	0.99–1.01
Gender			
Male	7.0	ref	
Female	9.6	1.41*	1.03–1.91
Marital status			
Married / Cohabit	9.0	Ref	
Single / Divorced / Separated / Widowed	8.0	1.13	0.84–1.54
Education level			
Tertiary or above	6.3	ref	
Secondary	7.2	1.15	0.74–1.79
Primary or below	12.6	2.15**	1.36–3.41
Occupation			
Skill levels 3 or 4	3.9	Ref	
Skill level 2	7.3	1.88*	1.01–3.51
Skill level 1	12.1	3.26***	1.73–6.17
Student	9.2	2.46*	1.06–5.68
Looking after family / home	9.6	2.54**	1.33–4.84
Income Poverty			
Non-poor	6.4	Ref	
Poor	19.4	3.52***	2.50–4.95
**Lifestyle factors**			
Smoking Status			
Non Smoker	7.9	Ref	
Past smoker / Current smoker	10.5	1.35	0.95–1.93
Alcohol Drinking			
Non-risky drinker	8.5	ref	
Risky drinker	7.4	0.89	0.39–2.04
Physical Activities			
Active	10.8	ref	
Minimally active	4.6	0.41*	0.21–0.82
Inactive	8.7	0.79	0.50–1.25
**Physical and mental health conditions**			
Number of chronic diseases			
0–1	7.5	ref	
≥ 2	12.2	1.72**	1.22–2.43
Body image			
About the right weight	7.2	Ref	
Negative body image	9.9	1.43*	1.06–1.93
Sleeping quality			
Very good / Fairly good	6.3	Ref	
Fairly bad / Very bad	13.9	2.37***	1.75–3.22
Interference by pain on daily activities			
No pain at all	3.1	Ref	
Not affected / A little bit affected	10.8	3.80***	2.47–5.83
Quite affected / Extremely affected	18.1	7.06***	4.31–11.55
High blood pressure			
No	8.0	ref	
Yes	7.7	0.96	0.66–1.40
BMI (kg/m^2^)			
Normal	7.4	ref	
Overweight	8.9	1.22	0.79–1.87
Obese	9.1	1.24	0.85–1.81
Underweight	5.6	0.75	0.37–1.53
Central Obesity			
No	6.9	ref	
Yes	10.2	1.56**	1.12–2.16
Disability (P1)			
No	4.4	ref	
Yes	14.4	3.65***	2.65–5.03
Disability (PM)			
No	5.8	Ref	
Yes	18.8	3.78***	2.77–5.15
SF-12v2 Physical Component Summary			
4^th^ quartile	5.7	Ref	
3^rd^ quartile	4.4	0.76	0.45–1.30
2^nd^ quartile	6.8	1.20	0.73–1.97
1^st^ quartile	18.7	3.79***	2.45–5.86
SF-12v2 Mental Component Summary			
4^th^ quartile	3.1	Ref	
3^rd^ quartile	5.9	1.94*	1.09–3.43
2^nd^ quartile	6.6	2.22**	1.25–3.92
1^st^ quartile	20.0	7.74***	4.67–12.84
Anxiety			
Normal	6.4	Ref	
Anxious	25.7	5.04***	3.57–7.13
Stress			
Normal	6.8	Ref	
Stressed	34.6	7.16***	4.82–10.65

*p<0.05.

**p<0.01.

***p<0.001.

Median monthly equivalised household income of those who were unable to seek medical care due to lack of financial means (HK$7,668) was lower than that of other participants (HK$12,500) (Table 3). Of the respondents who did not seek medical care due to lack of financial means, around one-fifth of them (20·5%) were on CSSA (data not shown in Table).

**Table 3:** Distribution of monthly equivalised household income for those having financial barriers to medical care.

	Median (interquartile range) / Row %
Equivalised household income (HK$)	7,668 (6,593)
Equivalised household income (in quartiles)^a^	
1st (lowest)	18.5
2^nd^	9.3
3^rd^	7.5
4th (highest)	2.3

^a^ Monthly equivalised household income was categorized into quartiles based on the distribution of the entire sample

All factors that were significantly associated with “not seeking medical care due to a lack of financial means” were included into a forward stepwise logistic regression (Table 4). In multiple regression analysis, participants who were income-poor (OR = 2·83, 95% CI = 1·85–4·32) were more likely not to seek medical care due to lack of financial means. While being minimally active had reversed significant association with having financial barriers to medical care, there was no clear trend across the physical activity levels. Having worse physical and mental health related quality of life (physical health: OR = 2·11, 95% CI = 1·17–3·81, 1^st^ quartile compared with 4^th^ quartile) (mental health: OR = 3·84, 95% CI = 1·94–7·59, 1^st^ quartile; OR = 2·27, 95% CI = 1·12–4·62, 3^rd^ quartile; compared with 4^th^ quartile), more severe pain symptoms that affected their daily activities (OR = 2·31, 95% CI = 1·39–3·83, not affected / a little bit affected; OR = 2·42, 95% CI = 1·29–4·52, not affected / a little bit affected; compared with no pain), greater disability (OR = 1·90, 95% CI = 1·24–2·91, presence of at least some difficulty in any domains), and higher levels of both anxiety and stress (anxiety: OR = 1·86, 95% CI = 1·08–3·21; stress: OR = 2·06, 95% CI = 1·09–3·89) were all independently associated with being unable to seek healthcare due to lack of financial means. Having multimorbidity, on the other hand, was significantly associated with lower likelihood of being unable to seek healthcare due to lack of means (OR = 0·56, 95% CI = 0·34–0·92, more than one chronic diseases compared with one or no diseases).

**Table 4:** Forward stepwise logistic regression model, adjusted odds ratio for having financial barriers to medical care.

	Adjusted OR	95% CI
Income Poverty		
Non-poor	ref	
Poor	2.83***	1.85–4.32
Physical Activities		
Active	Ref	
Minimally active	0.31**	0.14–0.71
Inactive	0.68	0.39–1.18
Number of chronic diseases		
0–1	Ref	
≥ 2	0.56*	0.34–0.92
Interference by pain on daily activities		
No pain at all	ref	
Not affected / A little bit affected	2.31**	1.39–3.83
Quite affected / Extremely affected	2.42**	1.29–4.52
Disability (P1)		
No	Ref	
Yes	1.90**	1.24–2.91
SF-12v2 Physical Component Summary		
4^th^ quartile	ref	
3^rd^ quartile	1.32	0.71–2.46
2^nd^ quartile	1.23	0.67–2.24
1^st^ quartile	2.11*	1.17–3.81
SF-12v2 Mental Component Summary		
4^th^ quartile	Ref	
3^rd^ quartile	2.27*	1.12–4.62
2^nd^ quartile	1.91	0.93–3.91
1^st^ quartile	3.84***	1.94–7.59
Anxiety		
Normal	Ref	
Anxious	1.86*	1.08–3.21
Stress		
Normal	Ref	
Stressed	2.06*	1.09–3.89

*p<0.05.

**p<0.01.

***p<0.001.

## Discussion

Although the policy goal of the Hong Kong government is to ensure that “no one shall be denied adequate medical care through lack of means”[10], we found that about 8% of the Hong Kong population reported that they were unable to access medical care due to lack of financial means. We also found that this group of people had higher health needs–i.e., they were generally sicker in terms of both physical and mental health when compared with people who did not face financial barriers to healthcare access.

The inverse care law[28,29] states that “the availability of good medical or social care tends to vary inversely with the need of the population served” and the law “operates more completely where medical care is most exposed to market forces, and less so where such exposure is reduced.” It is well established that there is a strong relationship between socio-economic factors and health outcomes[30]. Other studies have shown that poor social status is associated with poorer access to healthcare[31,32]. Delaying or forgoing medical treatments, largely due to financial reasons, were found to have negative impact on health both in terms of poorer self-related health and lower quality of life scores[33], which echoes our findings on poorer physical and mental health-related qualities of life among those who were unable to access medical care due to financial reasons. This results in the unjust situation that the poor have both worse health and lower access to medical services. As Hong Kong has a significant private sector especially for outpatient services, the operation of the inverse care law is likely.

Apart from general physical and mental health statuses, independent associations were found for other specific health conditions. Those who were unable to access healthcare due to financial reasons were less likely to have multimorbidity, and it is possibly the result of potential over-adjustments in the final model. People being minimally physically active was identified to be inversely associated to have financial barrier to medical care when comparing to those defined as active. However, there was no consistent trend across the three levels of physical activities and only the minimally physical active were significantly associated with having financial barrier to medical care. Our results are in contrast to findings from previous studies which demonstrated that low physical activity was strongly associated with poor financial condition [34] and was risk factor of multiple medical conditions [35,36]. Although we do not know the reasons for the inconsistent findings, we postulate that people with high physical activity may be people whose occupation involves manual labour with lower socio-economic status. The causality of the relationship can only be answered using future prospective study design.

Our study results showed that with the increase of interference by pain on daily activities, the odds of facing financial difficulty to medical care also increased. Previous studies suggested that people with chronic pain may have difficulty in seeking medical care due to limited financial resources for transportation and high cost associated with the treatment of unrelieved pain [37,38]. We also found that disability was positively associated with having financial barriers to access medical care. A study conducted among people with disability [39] has shown that the high association with the use of medical services (e.g. medications, medical equipments) were the main financial barriers to access medical care. The greater financial burden associated with these non-acute but long-term limiting illnesses may lead to medical impoverishment and thus a higher level of financial barriers to healthcare, as people with more severe long-term functional limitations, especially among disadvantaged groups, were more likely to experience greater economic hardship and financial strain[40–42].

Regarding mental conditions, our findings of the greater levels of both anxiety and stress among those denied of healthcare due to financial barriers echoed previous research supporting the relationship between cost-related avoidance of medical care and frequent mental distress[43]. These observations supported the notion that healthcare insecurity (i.e., uncertainty about the ability to access and sustain needed health services) is associated with poorer health-related quality of life and greater mental distress[44]. In contrast, other studies reported that being unable to seek healthcare with respect to lack of financial means might lead to poor health status [45–47]. As this is only a cross-sectional study, no causal relationship can be drawn. Therefore, we can only examine the causal relationship between identified associated factors with being unable to seek medical care due to lack of financial means in future prospective study.

In addition, previous studies have shown that in Hong Kong, the quality of primary care and accessibility is better among those with higher income or with private insurance[48,49] and that private primary care providers provide better accessibility and interpersonal continuity of care. This may have resulted in the overall effect that people with higher incomes have better accessibility and quality of primary care[49] while the poor have less access, despite the fact that poor people who are eligible for social assistance can receive medical services free of charge at the point of care. Furthermore, US research previously showed that up to 12% of the health deterioration associated with delaying or forgoing medical treatments could be avoided by an expansion of health insurance coverage [33], which may shed light on the potential responsibility of less affordable private primary care in generating inequalities in healthcare utilization.

Although Hong Kong public healthcare system is largely subsidized, population surveys conducted in other developed countries such as Australia, Canada, and New Zealand showed that reliance on private coverage to supplement public coverage could result in inequities in access to healthcare despite the provision of full coverage for basic healthcare[50]. A previous study conducted in Hong Kong showed that even a co-payment as low as US$8 can deter patients from using screening services[51]. Even though the public healthcare sector of Hong Kong is mainly tax-funded, and usage incurs only a small amount of co-payment, it is speculated that there will be people who are inadequately protected and thus unable to use medical services when needed because of these financial barriers. Another study has shown that household income per capita can determine healthcare utilization irrespective of healthcare needs, and reported a lower healthcare utilization among the less affluent elderly when compared with their richer counterparts[11].

At present, there is an ongoing Healthcare Voucher Scheme to increase supplemental coverage for elderly patients to purchase private medical services in Hong Kong. Whether this will result in an improved public-private balance in healthcare service usage or more inequities in access awaits further evaluation. Previous studies conducted in Hong Kong showed that amongst the elderly, the willingness-to-pay for private services such as specific primary care, chronic disease care, and preventive services is low[52]. The use of financial incentives such as government vouchers may encourage the elderly to visit private medical outpatient services for preventive and chronic care, although greater efforts may be needed to change the health seeking behaviour of the older population for public medical services[53].

There are several limitations in the present study. First, it is a cross-sectional study and no conclusion can be drawn about the temporal relationships between factors associated with being unable to seek medical care due to lack of financial means. Second, we have only used a single question in this analysis to define being unable to access medical care due to insufficient financial means over the previous 12-month period; and as with other self-reported surveys, recall bias may have affected the accuracy of the results. Third, residual confounding is possible as there may be unmeasured confounding factors associated with being unable to access medical care due to financial reasons (e.g. endogeneity). Fourth, we relied on participants’ self-reported need for medical care which may not be consistent with health professional’s assessment of their health needs. However, we have demonstrated that the people who have been financially excluded from access to healthcare are likely to have higher medical needs due to their higher prevalence of disability and pain, poorer quality of life, and greater reported mental health outcomes. Last, due to the nature of the question on medical access, people who did not have access problems in seeking medical care due to financial reasons may either be those who did not need to seek medical care or those who needed and were able to access healthcare. To address this limitation and ensure valid comparisons, we replicated the analyses in a subgroup of people with poorer self-rated health (68.1% of the final sample) who presumably have greater healthcare needs comparable to those prevented form medical care due to financial barriers. The results were similar between overall and subgroup analyses (S1 Table), which suggested the robustness of our findings even after the exclusion of people who had less healthcare needs.

Despite these limitations, the current findings are important in several ways. This is the first study in Hong Kong to examine the associations between patients’ reported lack of medical access and its potential determinants. The regular collection and reporting of such data in Hong Kong in the future would assist policy makers to better identify resource allocation priorities and to target the population who require additional help to access the medical care they need. More research in this area may enhance the understanding of this population group and their health seeking behaviours. As our results showed that not all of them were receiving social assistance with its eligibility mainly based on government’s assessments of income and assets, other factors related to their health-seeking behaviour is worth exploring in future research.

Many developing and developed countries are reforming their health systems to move towards UHC[1]. It is time for Hong Kong, which is one of the richest societies in the world, with the highest Gini coefficient[9], to consider improving access to quality healthcare services for the poor. In addition to CSSA as a safety net, further protections beyond endowment policies are necessary to improve medical access among the poor. Expanding the existing government-led Healthcare Voucher Scheme, specifically for older adults at present, to cover disadvantaged middle-aged adults might be an option. In the long run, strengthening of the publicly funded primary care system will be the key to achieving UHC in Hong Kong.

## Supporting information

S1 Table Forward stepwise logistic regression model for comparing those could not see doctors due to financial reasons and could see doctors, excluding those rated their health as excellent or very good. (DOCX)
