# Supplementary material for: What are the financial barriers to medical care among the poor, the sick and the disabled in the Special Administrative Region of China?
Source: PLoS One. 2018 Nov 14;13(11):e0205794. doi: 10.1371/journal.pone.0205794 (PMC6235271; doi:10.1371/journal.pone.0205794)
Supplement: S1 Table — (DOCX) [file pone.0205794.s001.docx]

**Supplementary Table 1. Forward stepwise logistic regression model for comparing those could not see doctors due to financial reasons and could see doctors, excluding those rated their health as excellent or very good**

|  | Adjusted OR | 95% CI |
| --- | --- | --- |
| Income Poverty |  |  |
| Non-poor | ref |  |
| Poor | 2.49*** | 1.60 – 3.87 |
| Physical Activities |  |  |
| Active | ref |  |
| Minimally active | 0.26** | 0.10 – 0.66 |
| Inactive | 0.66 | 0.35 – 1.24 |
| Interference by pain on daily activities |  |  |
| No pain at all | ref |  |
| Not affected / A little bit affected | 2.80*** | 1.55 – 5.05 |
| Quite affected / Extremely affected | 3.01** | 1.55 – 5.86 |
| Disability (P1) |  |  |
| No | ref |  |
| Yes | 1.89** | 1.22 – 2.92 |
| SF-12v2 Mental Component Summary |  |  |
| 4^th^ quartile | ref |  |
| 3^rd^ quartile | 1.65 | 0.72 – 3.77 |
| 2^nd^ quartile | 1.29 | 0.58 – 2.88 |
| 1^st^ quartile | 2.83** | 1.33 – 6.01 |
| Anxiety |  |  |
| Normal | ref |  |
| Anxious | 1.93* | 1.11 – 3.38 |
| Stress |  |  |
| Normal | ref |  |
| Stressed | 2.06* | 1.09 – 3.88 |

*p<0.05, **p<0.01, ***p<0.001
